# Effectiveness in Remineralizing White Spot Lesions in Primary Teeth With Varnishes: A Systematic Review

**DOI:** 10.1002/cre2.70403

**Published:** 2026-07-08

**Authors:** Carolina Caleza‐Jiménez, María Biedma‐Perea, David Ribas‐Pérez, Maria José Barra‐Soto, Marcela Arenas‐González

**Affiliations:** ^1^ Department of Stomatology University of Seville Seville Spain

**Keywords:** fluoride varnish, primary teeth, remineralization, white spot lesion

## Abstract

**Objectives:**

This systematic review aims to assess the effectiveness of varnishes for the remineralization of white spot lesions in primary teeth.

**Methods:**

A database search was conducted in MEDLINE and the Cochrane Library to screen for articles relevant to the topic of the review. Risks of bias in randomized studies were assessed using the ROB2 tool and GRADE evidence levels.

**Results:**

Seven studies met all inclusion criteria and were included. Differences in varnish composition and characteristics can affect the anticaries properties. Bioactive additives had a statistically significant reduction in ICDAS scores.

**Conclusions:**

Intensive application of remineralizing varnishes demonstrated a significant effect on the remineralization and control of carious lesion activity. New‐generation fluoride appear to be more effective than conventional sodium fluoride varnishes.

## 1 Introduction

The American Academy of Pediatric Dentistry (AAPD) defines early childhood caries (ECC) as the presence of one or more carious lesions (cavitated or non‐cavitated), missing teeth due to caries, or fillings in any primary tooth in children aged 71 months (Sohn et al. 2006). This pathology is a global problem that affects all children worldwide (Tinanoff 2005). Caries lesions are acute diseases that require lesion cleaning, cavity preparation, and tooth restoration over time. However, an increasing number of practitioners are using individually tailored strategies to prevent, arrest, or improve the disease process based on the existing risk of caries (Council on Clinical Affairs 2018; Academy et al. 2018).

The first affected teeth are the upper incisors since during sucking, the “teat” rests on the palate and the flow on the drink directly touches the palatal surfaces of these teeth (Hallett and O'Rourke 2006). They are extensive and multiple lesions, which progress very quickly, causing cavitation and destroying the crown of the teeth; hence, a quick and effective treatment is required. Although the lesions initially do not cause any discomfort, it will observe pain, repeated infections, and even weight loss when eating as the lesion process advances to more dental structure (Finlayson et al. 2007).

The International Caries Diagnosis and Detection System (ICDAS) is a technique for diagnosing caries lesions that is proposed to reduce subjectivity and increase specificity and sensitivity, thereby allowing the reproducibility of visual tactile inspection in caries diagnosis (Ismail et al. 2007). The high sensitivity of this detection system to each of these codes has allowed the design of treatment protocols applicable to temporary and permanent dentition (Clara et al. 2012), from the placement of fluoride in varnish as a remineralizing element to the placement of direct and indirect restorative materials.

Minimally invasive treatments are currently advocated, requiring less chair time and causing less anxiety in children. Hence, it is important to understand how these lesions can be detected and managed in the early stages, allowing for the application of noninvasive treatments. Treatment of the affected surface with codes 1 and 2 is primarily based on the placement of remineralizing agents (Patel et al. 2017). Among the therapies, varnish is the most suitable for this age group as it can be applied quickly, causing less patient discomfort and hence greater patient acceptability without esthetic changes (Ismail et al. 2007). However, the caries‐inhibiting effect of the different varnish on primary teeth is still debatable.

In light of the persistent global burden of ECC and the expanding use of minimally invasive, nonoperative approaches—particularly varnish‐based therapies—this systematic review was conducted to evaluate the effectiveness of fluoride varnishes to reduce white spots on primary teeth.

## 2 Methods

### 2.1 Protocol and Registration

This systematic review was conducted in accordance with the Preferred Reporting Items for Systematic Reviews and Meta‐Analyses (PRISMA) 2020 guidelines (Page et al. 2021), and was prospectively registered in the International Prospective Register of Systematic Reviews (PROSPERO) under protocol number ID1157516.

### 2.2 Review Question

The central research question of this systematic review was as follows: In children with primary dentition presenting white spot lesions, is the application of fluoride varnish more effective than other remineralizing agents in promoting lesion remineralization?

This question was developed using the PICOS framework (Hoogendam et al. 2012):

- **Population**: Children under 6 years with lesions ICDAS codes 1 or 2
- **Intervention**: Any form of fluoride varnish (professionally applied) used for the prevention of ECC
- **Comparators**: Any other form or concentration of varnish, placebo, or no intervention
- **Outcomes**: Remineralization of white spot lesions

### 2.3 Eligibility Criteria

Inclusion and exclusion criteria were established according to the Strength of Recommendation Taxonomy (SORT) guidelines. Table 1 provides a detailed overview of these criteria.

**Table 1:** Inclusion and exclusion criteria.

Inclusion criteria	Exclusion criteria
Studies published in the last 10 years	Case series, reviews, letters, and commentaries
Studies evaluating the effectiveness of varnishes in remineralizing white spot lesions in primary teeth	Studies not related to the objective of this review
Papers written in English	
Studies with a sample size of at least 30 children	

### 2.4 Search Strategy

A comprehensive literature search was conducted using PubMed and Cochrane Library databases. The following terms were used in the search: “minimally invasive surgical procedures,” “calcium phosphates,” “fluorides,” “child,” and “dental caries.” Medical Subject Heading (MeSH) terms were strategically combined using the Boolean operators “AND” and “OR” to optimize search specificity and sensitivity.

### 2.5 Selection of Studies

We were screened to exclude clearly irrelevant studies. Abstracts of the remaining studies were reviewed in the second phase to eliminate non‐eligible references. A manual search of references was also performed at this stage. The full‐text articles from the remaining studies were evaluated for inclusion in the final phase. Discrepancies between reviewers were elucidated through discussion until a consensus was reached. Owing to clinical and methodological heterogeneity, a quantitative meta‐analysis was not feasible. To assess the risk of bias of eligible studies, the reviewers used the Cochrane Risk of Bias tool 2.0 (Sterne et al. 2019).

### 2.6 Analysis of GRADE Evidence Levels

We graded the certainty of evidence of relevant outcomes based on current GRADE (Grading of Recommendations Assessment, Development and Evaluation) guidance (Guyatt et al. 2008). This approach incorporates five key domains: (1) Risk of bias, (2) inconsistency, (3) indirectness, (4) imprecision of the evidence, and (5) reporting bias. Two reviewers assessed each domain for each selected outcome and resolved differences by consensus discussion.

## 3 Results

### 3.1 Study Selection

**Figure 1:**
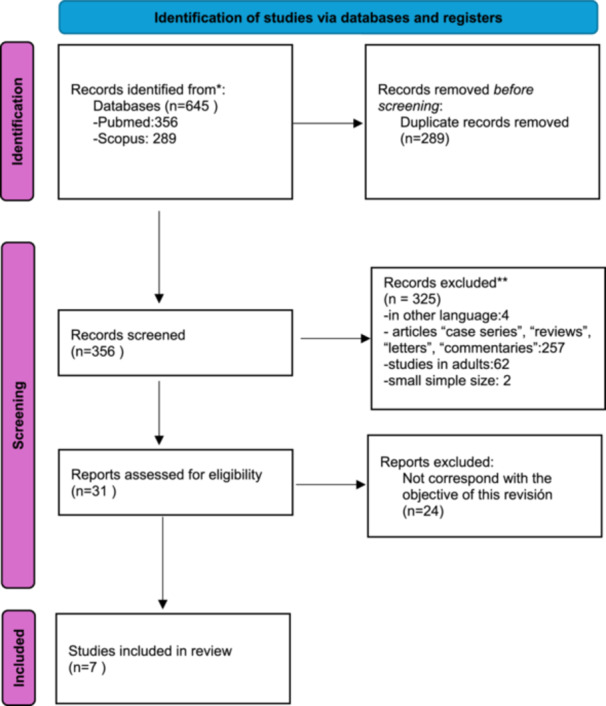
PRISMA flow diagram illustrating the study selection and screening process.

Figure 1 presents the selection process. Following the initial database search, 645 records were identified. Before screening, we removed 289 duplicate records. After applying the defined inclusion criteria, 31 articles were selected for further evaluation. Of these studies, 24 were excluded because they do not correspond to the objective of this revision. Ultimately, seven studies met all inclusion criteria and were included in the present systematic review (Mekky et al. 2021; Turska‐Szybka et al. 2021; Manchanda et al. 2023, 2024; Natchiyar et al. 2023; Khairy, Talaat, Essa et al. 2025).

### 3.2 Analysis of Included Studies

Table 2 summarizes the data extracted from each study, and the Table 3 contains the risk of bias of eligible studies.

**Table 2:** Summarizes the key characteristics of the seven included studies.

Author, year	Sample	ICDAS code	Follow‐up	Groups	Intensive protocol of application	Conclusions
Mekky et al. (2021)	44 c/3–5 y	2	7 m	(G1) MI Varnish (G2) Duraphat	After 2 w (3 sessions)	CPP‐ACP with NaF was more effective than NaF in white spot lesions mineralization.
Turska‐Szybka et al. (2021)	180 c/3–6 y	NR	12 m	(G1) Fluor Protector S (G2) Duraphat (G3) OHT and cleaning	Every 3 m (4 sessions)	The 2 fluoride varnishes demonstrated an equal capacity to reduce the incidence of caries in caries‐active preschool children over a 12‐month period in comparison with OHT and cleaning.
Manchanda et al. (2023)	135 c/3–4 y	2	24 m	(G1) Duraphat (G2) Clinpro White (G3) MI Varnish	Every 3 m (8 sessions)	CPP‐ACP with NaF varnishes demonstrated similar antibacterial effect on *S. mutans* and *L. fermentum* compared to conventional NaF varnish.
Natchiyar et al. (2023)	30 c/3–5 y	1/2	6 m	(G1) Embrace Varnish (G2) Curodont Repair	Only once	Self‐assembling peptide P11‐4, combined with NaF and xylitol‐coated calcium and phosphate with NaF are effective at remineralizing white spot lesions in primary teeth and can be considered as remineralizing agents.
Manchanda et al. (2024)	381 c/3–4 y	2	24 m	(G1) Duraphat (G2) Clinpro White (G3) MI Varnish	Every 3 m (8 sessions)	CPP‐ACP with NaF showed similar efficacy against cavitated and non‐cavitated carious lesions as compared to conventional NaF in high‐risk preschool children
Khairy, Talaat, Essa et al. (2025)	66 c/3–6 y	1/2	3 m	(G1) Curodont Repair (G2) MI Varnish (G3) Duraphat	After 2 and then after 4 w (3 sessions)	Self‐assembling peptide P11‐4 combined with sodium fluoride presented a superior antibacterial effect when compared to CPP‐ACP with NaF or NaF, which exhibited a comparable antibacterial effect.
Khairy, Talaat, Dowidar et al. (2025)	66 c/3–6 y	1/2	12 m	(G1) Curodont Repair (G2) Varnish (G3) Duraphat	After 2 and then after 4 w (3 sessions)	Intensive application of self‐assembling peptide P11‐4 combined with NaF exhibited superior remineralization and esthetic improvement of white spot lesions compared to CPP‐ACP with NaF or NaF

Abbreviations: c, children; CPP‐ACP, Casein phosphopeptide amorphous calcium phosphate fluoride; G, group; m, months; NaF, sodium fluoride; NR, not registered; OHT, oral hygiene teach; w, weeks; y, years.

**Table 3:** Risk of bias for the seven studies included.

Study	Specimen randomization	Single operator	Operator blinded	Control group	Standardized specimens	Outcome mode	Manufacturer's instructions	Sample size calculation	ROB
Mekky et al. (2021)	Yes	No	Yes	No	Yes	Yes	Yes	Yes	Moderate
Turska‐Szybka et al. (2021)	Yes	No	Yes	Yes	No	Yes	Yes	Yes	Moderate
Manchanda et al. (2023)	Yes	Yes	Yes	No	Yes	Yes	Yes	Yes	Low
Natchiyar et al. (2023)	Yes	No	Yes	No	Yes	Yes	Yes	Yes	Moderate
Manchanda et al. (2024)	Yes	Yes	Yes	No	Yes	Yes	Yes	Yes	Low
Khairy, Talaat, Essa et al.(2025)	Yes	No	Yes	No	Yes	Yes	Yes	Yes	Moderate
Khairy, Talaat, Dowidar et al. (2025)	Yes	No	Yes	No	Yes	Yes	Yes	Yes	Moderate

*Note:* A score of 7–8 indicates low risk, 4–6 indicates some concern, and 1–3 indicates high risk.

#### 3.2.1 Duraphat Versus Fluor Protector S

Turska‐Szybka et al. (2021) observed that both 1.5% ammonium fluoride varnish (Fluor Protector S) and 5% sodium fluoride (NaF) varnish (Duraphat) reduced the incidence of caries than the control group; however, no statistically significant difference was found between the two. Relative to the control group, the relative risk of developing cavitated lesions was 0.39 with Fluor Protector S and 0.26 with Duraphat. The certainty of the evidence was rated as moderate.

#### 3.2.2 Duraphat Versus MI Varnish

Casein phosphopeptide‐amorphous calcium phosphate (CPP‐ACP), incorporated into a 5% NaF varnish—forming the CPP‐ACP NaF varnish (MI Varnish)—was investigated by Mekky et al. (2021) in comparison to Duraphat. The MI Varnish intervention resulted in a significantly greater reduction in the number of active lesions than Duraphat. The certainty of the evidence was rated as moderate.

#### 3.2.3 Duraphat Versus MI Varnish or Clinpro White

Tricalcium phosphate (TCP), incorporated into a 5% NaF varnish—forming the TCP‐ NaF varnish (Clinpro White)—has also been evaluated and compared with Duraphat and MI Varnish. In a 2023 study, Manchada et al. (2023). assessed the in vivo antibacterial effect on *Streptococcus mutans* (SM) and *Lactobacillus fermentum* (LF). A statistically significant difference was observed in the percentage of detectable biofilm LF and salivary SM. Clinpro White exhibited a significant increase in the percentage of children with salivary SM, salivary LF, and biofilm LF. Similarly, with MI Varnish, a significant increase was observed in salivary LF. The certainty of the evidence was rated as high.

More recently, in 2024 (Manchanda et al. 2024), the incidence of new caries over a 2‐year period was reported as follows: 59.2% in the MI Varnish group, 65.1% in the Clinpro White group, and 66.1% in the Duraphat group, with no statistically significant difference. Similarly, the mean increment in cavitated lesions did not differ significantly among the three groups. The certainty of the evidence was rated as high.

#### 3.2.4 Duraphat Versus MI Varnish or Curodont Repair

Another varnish studied has been Curodont Repair, which introduces the P11‐4 self‐assembling peptide combined with 0.05% NaF. Khairy, Talaat, Essa et al. (2025) observed that all study groups exhibited a significant decrease in SM counts. Multivariable linear regression demonstrated a significantly greater reduction in SM counts with MI Varnish and Duraphat than Curodont Repair. MI Varnish showed superior bacterial reduction than Duraphat and Curodont Repair, with no significant difference between the latter two materials. Plaque index was significantly reduced in all study groups, with MI Varnish proving to be the most effective. The certainty of the evidence was rated as moderate.

In a 2025 study by Khairy, Talaat, Dowidar et al. (2025), it was found that Curodont Repair achieved significantly higher odds of caries arrest and a greater reduction in the number of active lesions than Duraphat. No significant differences were observed between MI Varnish and Duraphat regarding caries arrest or lesion activity reduction. The certainty of the evidence was rated as moderate.

#### 3.2.5 Curodont Repair Versus Embrace Varnish

Embrace Varnish, which contains 5% NaF and xylitol‐coated calcium and phosphate, was compared with Curodont Repair in the study by Natchiyar et al. (2023). They observed a statistically significant reduction in ICDAS scores and in the percentage area of WSLs with Curodont Repair. No statistically significant changes were found with Embrace Varnish. The certainty of the evidence was rated as low.

## 4 Discussion

As recommended by the AAPD (4), professionally applied fluoride varnish to primary teeth at least twice per year is highly advised to reduce the risk of ECC. Clinical studies (Marinho et al. 2013) have demonstrated a moderate level of effectiveness for fluoride varnish application in preventing dental caries in children (Weintraub et al. 2006). Intensive application has been suggested as a method to prolong fluoride contact time with the enamel surface (Croll and Berg 2014). Differences in varnish composition and characteristics can affect the anticaries properties of these products (Dehailan et al. 2019). In the last 5 years, studies comparing different varnishes have been conducted and evaluated in this systematic review.

Fluor Protector S has been analyzed in the study by Turska‐Szybka et al. (2021). Both Fluor Protector S and Duraphat significantly reduced caries incidence compared to the control group, but no significant differences were found between the two varnish formulations. However, the performance of the 1.5% ammonium fluoride varnish in primary dentition has not been previously reported.

On the other hand, CPP‐ACP, a nanocomplex of milk protein and amorphous calcium phosphate, is capable of remineralizing the deeper layers of WSLs (Cochrane et al. 2010). CPP‐ACP has been incorporated into NaF varnish, forming CPP‐ACP NaF varnish (MI Varnish). The study included in this systematic review by Mekky et al. (2021) showed that both varnishes were effective in remineralizing WSLs in primary teeth; however, intensive application of MI Varnish was superior to Duraphat. These findings align with those of Attiguppe et al. (2019), who demonstrated that MI Varnish has greater antimicrobial efficacy against SM than Duraphat, resulting in reduced plaque formation. Another recently introduced varnish containing TCP in addition to 5% sodium fluoride (Clinpro White) has been reported to be effective in preventing ECC among preschool children (Colombo et al. 2017). In the study of this systematic review, Manchada et al. (2024) found that MI Varnish and Clinpro White demonstrated similar efficacy in caries prevention when compared to Duraphat. The findings from this study differ from those of Mekky et al. (2021). These discrepancies may be attributed to diagnostic criteria or frequency of applications.

However, the in vivo antibacterial effects of these novel agents on SM and LF have not been extensively investigated. In the study by Manchada et al. (2024), included in this review, both MI Varnish and Clinpro White demonstrated similar effects on SM and LF counts when compared with Duraphat. A similar study (Sajjan et al. 2013) also failed to show a significant decrease in biofilm SM levels following application Duraphat. In contrast, a randomized clinical trial (9) demonstrated that a fluoride varnish containing additional CPP‐ACP significantly reduced salivary SM levels. These divergent results may be attributed to differences in follow‐up duration, application frequency, and participant age groups.

In the study included in this revision by Khairy, Talaat, Essa et al. (2025), they compared the antibacterial effect of different varnish against SM. The results of this study demonstrated that all study groups resulted in a significant reduction in SM count. It was found that MI Varnish presented more antibacterial effect against SM than P11‐4 with fluoride varnish (Curodont Repair). These results come in partial accordance with a recent study by Atteya et al. (2024) who demonstrated a significant reduction in SM count in subjects treated with P11‐4 in comparison to those treated with NaF. The notable antibacterial effect of P11‐4 with fluoride was confirmed by an in vitro study, which was done by El‐Kaddah et al. (2024), who demonstrated the formation of inhibition zones on agar plates cultured with SM and treated with P11‐4 with fluoride.

On the other hand, the study by Khairy, Talaat, Dowidar et al. (2025) present review, revealed that treatment with Curodont Repair resulted in a superior caries arrest effect compared to Duraphat. These findings are consistent with those reported by Alkilzy et al. (2018), who observed that a single application of Curodont Repair in combination with 5% NaF varnish was more effective than 5% NaF varnish alone in arresting early carious lesions on permanent teeth. Similarly, Shalaan et al. (2024) demonstrated that a single application of Curodont Repair to WSLs significantly reduced compared to Duraphat. This enhanced effect may be explained by the ability of P11‐4, the active peptide in Curodont Repair, to deeply infiltrate the body of early carious lesions, a mechanism supported by several in vitro studies (Kind et al. 2017).

Curodont Repair was also selected in the study by Natchiyar et al. (2023) to evaluate its effectiveness in the management of WSLs, in comparison with Embrace Varnish. The findings revealed that ICDAS scores were significantly reduced from baseline with Curodont Repair, whereas no statistically significant reduction was observed with Embrace Varnish. This suggests that Curodont Repair may exert a superior remineralizing effect on WSLs. While both groups exhibited reductions in ICDAS scores, only Curodont Repair demonstrated statistically significant improvements. Similar findings were reported by Alkilzy et al. (2018) and Kobeissi et al. (2020). Conversely, Shahmoradi et al. (2017) reported a decrease in enamel demineralization after the application of Embrace Varnish, supporting its potential, albeit to a lesser extent.

There are several limitations that must be acknowledged. First, detailed assessments of dietary habits and oral hygiene practices were not consistently reported across the included studies, limiting the ability to evaluate their potential influence. Another notable limitation is the variability in the time intervals between applications. It is essential to evaluate the long‐term effects of the studied materials, as most studies included in this review had relatively short follow‐up periods. In addition, the absence of a proper control group in many studies hinders the ability to definitively attribute remineralization effects to the intervention alone. To address these limitations, future research should focus on conducting randomized controlled trials with larger sample sizes and clearly defined control groups, as well as incorporating standardized protocols for application intervals and long‐term follow‐up assessments.

## 5 Conclusions

Within the limitations of the included studies, the following conclusions can be drawn:

- The intensive application of remineralizing varnishes demonstrated a significant effect on the remineralization and control of carious lesion activity in WSLs.
- New‐generation fluoride varnishes containing bioactive additives (CPP‐ACP, TCP, P11‐4) appear to be more effective reduction in active WSLs and superior esthetic outcomes when compared to conventional NaF varnishes.
- The use of fluoride varnishes should be considered a first‐line, non‐invasive treatment for managing early carious lesions (ICDAS 1–2) in primary teeth, combined with regular follow‐up, oral hygiene reinforcement, and dietary counseling.
- Long‐term follow‐up studies with larger sample sizes, greater statistical power, and well‐defined control groups are necessary in order to establish evidence‐based protocols for the use of varnishes

## Author Contributions

Conceptualization: Carolina Caleza‐Jiménez, María Biedma‐Perea, and David Ribas‐Pérez. Methodology: Carolina Caleza‐Jiménez, M. José Barra‐Soto, and Marcela Arenas‐González. Data curation: Carolina Caleza‐Jiménez and María Biedma‐Perea. Validation: all authors. Writing − original draft preparation: Carolina Caleza‐Jiménez, David Ribas‐Pérez, and Marcela Arenas‐González. Writing − review and editing: all authors. All authors have read and agreed to the published version of the manuscript.

## Funding

The authors have nothing to report.

## Consent

The authors have nothing to report.

## Conflicts of Interest

The authors declare no conflicts of interest.

## Data Availability

Data sharing not applicable to this article as no datasets were generated or analyzed during the current study.
